# Nicorandil Prevents Right Ventricular Remodeling by Inhibiting Apoptosis and Lowering Pressure Overload in Rats with Pulmonary Arterial Hypertension

**DOI:** 10.1371/journal.pone.0044485

**Published:** 2012-09-07

**Authors:** Xiang-Rong Zuo, Qiang Wang, Quan Cao, Yan-Zhe Yu, Hui Wang, Li-Qing Bi, Wei-Ping Xie, Hong Wang

**Affiliations:** 1 Department of Respiratory Medicine, The First Affiliated Hospital of Nanjing Medical University, Nanjing, Jiangsu, People’s Republic of China; 2 Department of Critical Care Medicine, The First Affiliated Hospital of Nanjing Medical University, Nanjing, Jiangsu, People’s Republic of China; Virginia Commonwealth University Medical Center, United States of America

## Abstract

**Background:**

Most of the deaths among patients with severe pulmonary arterial hypertension (PAH) are caused by progressive right ventricular (RV) pathological remodeling, dysfunction, and failure. Nicorandil can inhibit the development of PAH by reducing pulmonary artery pressure and RV hypertrophy. However, whether nicorandil can inhibit apoptosis in RV cardiomyocytes and prevent RV remodeling has been unclear.

**Methodology/Principal Findings:**

RV remodeling was induced in rats by intraperitoneal injection of monocrotaline (MCT). RV systolic pressure (RVSP) was measured at the end of each week after MCT injection. Blood samples were drawn for brain natriuretic peptide (BNP) ELISA analysis. The hearts were excised for histopathological, ultrastructural, immunohistochemical, and Western blotting analyses. The MCT-injected rats exhibited greater mortality and less weight gain and showed significantly increased RVSP and RV hypertrophy during the second week. These worsened during the third week. MCT injection for three weeks caused pathological RV remodeling, characterized by hypertrophy, fibrosis, dysfunction, and RV mitochondrial impairment, as indicated by increased levels of apoptosis. Nicorandil improved survival, weight gain, and RV function, ameliorated RV pressure overload, and prevented maladaptive RV remodeling in PAH rats. Nicorandil also reduced the number of apoptotic cardiomyocytes, with a concomitant increase in Bcl-2/Bax ratio. 5-hydroxydecanoate (5-HD) reversed these beneficial effects of nicorandil in MCT-injected rats.

**Conclusions/Significance:**

Nicorandil inhibits PAH-induced RV remodeling in rats not only by reducing RV pressure overload but also by inhibiting apoptosis in cardiomyocytes through the activation of mitochondrial ATP-sensitive K^+^ (mitoK_ATP_) channels. The use of a mitoK_ATP_ channel opener such as nicorandil for PAH-associated RV remodeling and dysfunction may represent a new therapeutic strategy for the amelioration of RV remodeling during the early stages of PAH.

## Introduction

Pulmonary arterial hypertension (PAH) is defined as a mean pulmonary artery pressure (mPAP) greater than 25 mmHg at rest with a pulmonary capillary wedge pressure, left atrial pressure, or left ventricular (LV) end-diastolic pressure less than or equal to 15 mmHg and a pulmonary vascular resistance (PVR) greater than 3 Wood units [1]. Severe PAH can cause death by progressively increasing PVR, which promotes right ventricular (RV) overload, pathological remodeling, dysfunction, and heart failure. The median survival time for patients with PAH is 2.8 years if left untreated. At present, prostanoids, endothelin-1 receptor antagonists, and phosphodiesterase-type 5 (PDE-5) inhibitors are used to improve the hemodynamics and the quality of life of patients with PAH [1]. However, these drugs produce only limited delays in the progress ion of PAH [2].

RV failure is the cause of at least 70% of deaths attributable to PAH [3]. The degree of RV remodeling is an independent prognostic indicator [4]. Many studies have confirmed that RV function is independently associated with prognosis of PAH [4], [5], [6], [7], [8]. Preventing and reversing RV remodeling and failure together with reducing pulmonary artery pressure (PAP) are therefore viable strategies for the treatment PAH [9], [10].

Unlike that of LV remodeling, the pathophysiology of RV remodeling is not well understood, such that treatments successfully in the case of LV remodeling often have no beneficial effect on RV remodeling. Clinical and experimental evidence suggest that the mechanical stress created by elevated pressure on the pulmonary artery is not the only cause of PAH-induced RV remodeling and failure [11], [12]. Some patients with severe PAH rapidly progress to RV failure but other patients do not [13]. RV myocardial function can also be impaired by factors such as sarcoidosis, scleroderma, and amyloidosis. These are potential contributing molecular mechanisms of RV remodeling independent of RV afterload [13]. For these reasons, the mechanisms underlying the development of RV hypertrophy (RVH) and remodeling merit further investigation.

Apoptosis plays an important role in the pathogenesis of LV remodeling. Inhibition of myocyte death is a viable therapeutic strategy [14]. In contrast, the development of PAH-induced RV remodeling and cardiomyocyte apoptosis is largely unknown. Recently, Maria et al. found that apoptosis plays a role in the progression of RV disease by using serial *in vivo* 99mTc-annexin scintigraphy [15]. This study may provide new insight into the course of cardiac cell apoptosis during RV remodeling and may aid in determining the optimal timing of antiapoptotic therapy to prevent or reverse RV remodeling.

Nicorandil, a mitochondrial ATP-dependent potassium (mitoK_ATP_) channel opener, has been shown to be cardioprotective. Nicorandil can inhibit the development of monocrotaline (MCT)-induced PAH by reducing PAP and RVH [16]. These effects may be associated with up-regulation of lung eNOS protein, improvement in pulmonary vascular endothelial activation, and anti-inflammatory and anti-proliferative effects on lung tissue. Nicorandil can also inhibit cardiomyocyte apoptosis induced by oxidative stress and hypoxia [17], [18], [19], and prevent LV remodeling [20], [21], [22]. However, whether nicorandil can inhibit RV cardiomyocyte apoptosis and prevent RV remodeling is still unknown.

The purpose of this study is to investigate the influence of nicorandil on PAH-induced RV remodeling and the potential mechanisms. To determine whether the effects of nicorandil on RV remodeling are due to the activation of mitoK_ATP_ channels, these effects were also investigated in the presence of 5-hydroxydecanoate (5-HD), a mitoK_ATP_ channel blocker. MCT injection for three weeks caused pathological RV remodeling, characterized by hypertrophy, fibrosis, dysfunction, and RV mitochondrial impairment, as indicated by increased levels of apoptosis. Nicorandil improved RV function and prevented maladaptive RV remodeling in rats with MCT-induced severe PAH. The protective effects of nicorandil on PAH-induced RV remodeling were found to be mediated not only by reduction of RV systolic pressure (RVSP) but also by inhibition of apoptotic cell death through opening of mitoK_ATP_ channels.

## Materials and Methods

### Ethics Statement

All animal experiments were carried out according to the National Institutes of Health Guide for the Care and Use of Laboratory Animals (publication no. 85-23, revised 1996) and approved by the Animal Care and Use Committee of Nanjing Medical University (Approval ID 2010-SR-001).

### *In vivo* model of PAH

Male Sprague-Dawley (SD) rats (200±20 g) were provided by the Sino-British SIPPR/BK Laboratory Animal Center (Shanghai, China). All animals were housed in groups of five per cage under standard laboratory conditions with free access to food and water at a constant room temperature of 22°C, 50–60%humidity, and a 12:12 day-night cycle. The rats were allowed to acclimate for 7 days before experimental procedures began.

Seventy-two rats received a single dose of 60 mg/kg of MCT injected intraperitoneally (ip). They were then randomly assigned to the following treatment groups: 0.9% saline (MCT-treated group), 7.5 mg·kg^−1^·d^−1^ nicorandil (nicorandil-treated group), and 5 mg·kg^−1^·d^−1^ 5-HD in combination with 7.5 mg·kg^−1^·d^−1^ of nicorandil (nicorandil and 5-HD-treated group). Treatments lasted 3 weeks. The age- and weight-matched control animals served as controls (control group) and received 0.9% saline. The doses of nicorandil and 5-HD used in this study were selected after review of previous studies [16], [23]. MCT (Sigma-Aldrich, St. Louis, MO, U.S.) was dissolved in 1 N HCl neutralized with 1 N NaOH and diluted with 0.9% NaCl. Nicorandil (Tokyo Chemical Industry Co., Ltd, Tokyo, Japan) and 5-HD (Sigma) were dissolved in saline concentrations of 3 mg/ml and 2 mg/ml, respectively. All treatments were administered orally by gavage once daily for 3 weeks.

Body weight (BW) was measured twice a week to adjust the dose accordingly. At the end of each week of the study, 5–8 rats per group were randomly selected for a designated experimental protocol.

### Hemodynamic Measurements

Twenty-one days after MCT/saline injection, all surviving rats were anesthetized with urethane (1.0 g/kg, ip) and measured for RVSP by right heart catheterization. Subcutaneous needle electrodes were inserted in the limbs for electrocardiogram (ECG) recording. The right jugular vein was isolated, and a small polyethylene catheter (PE10, Becton Dickinson, U.S.) was passed via a small transverse cut and then advanced into the RV under the guidance of the pressure tracing. After 20 minutes of stabilization, RVSP was recorded using a miniature pressure transducer (TSD104A,BIOPAC Systems, Inc., U.S.) digitized by a BIOPAC MP100 data acquisition system, and stored on a Dell PC. The femoral artery was cannulated with a small polyethylene catheter (PE 50, Becton Dickinson) for recording blood pressure (BP) with a transducer. The catheters were filled with heparinized saline (10 U/ml of heparin in 0.9% saline). ECG, RVSP and BP were simultaneously recorded on a polygraph. After hemodynamic measurements were completed, heparinized blood was collected for measurement of plasma brain natriuretic peptide (BNP). Whole lungs and hearts were also excised for Western blotting and histological analysis.

### Assessment of Right Ventricular Hypertrophy

After the atria, pulmonary trunk and aorta were removed from the excised heart, the RV wall was separated from the LV wall and ventricular septum. Wet weights of the RV, free LV, and ventricular septum were determined. The ratio of RV weight to body weight (RV/BW) and the ratio of the RV weight to LV plus septum weight (RV/[LV+S]) were calculated for assessment of RVH [24].

### Morphological Measurements

After being weighed, the RV was fixed with 10% neutral formalin (pH 7.40), embedded in paraffin, sectioned at a thickness of 4 µm, and stained with hematoxylin and eosin (HE) or Masson’s Trichrome staining as needed. The cross-sectional area (CSA) of cardiomyocytes and the extents of interstitial and perivascular fibrosis were observed as described previously [9].

### Ultrastructural Analysis

After being weighed, RV wall tissues (about 1 mm^3^) were fixed in 2.5% cold glutaraldehyde solution (pH 7.40) for 3 hours, rinsed with 0.1 mol/l phosphate-buffered saline (PBS), and immersed in 1% osmium tetroxide for additional 2 hours at room temperature. Tissues were then dehydrated through a graded series of ethanol to propylene oxide solutions and embedded in epoxy resin. Ultrathin sections were cut and then visualized under transmission electron microscope (JEM-1010, JEOL, Japan).

### TUNEL Assay

Detection of cardiomyocyte apoptosis was performed using a terminal transferase dUTP nick end labeling (TUNEL) assay kit (In Situ Cell Death Detection Kit, Fluorescein; Roche Diagnostics, Germany) to detect apoptotic cell nuclei. 4′, 6-diamidino-2-phenylindole (DAPI) (Sigma) was used to stain all cell nuclei. Briefly, sections were deparaffinized, digested with proteinase K (20 µg/ml) at room temperature for 15 minutes, and soaked in PBS for 5 minutes. Each section was then incubated with 50 µl of the TUNEL reaction mixture containing terminal deoxynucleotidyl transferase for 1 hour at 37°C in a dark, humidified chamber. Then slides were rinsed in water and counterstained with DAPI for cell nuclei. Sections incubated with PBS instead of TdT enzyme solution served as the negative controls. After two washes, the fluorescence images were observed and captured using a confocal microscope (Zeiss LSM510, Oberkochen, Germany) at the same magnification (×400). The rate of apoptosis is here expressed as a ratio of the number of TUNEL-positive myocytes to the total number of myocytes stained with DAPI from three different random fields per heart (n = 3). All of these assays were performed blind. Four rats from each group were evaluated.

### Enzyme-linked Immunosorbent Assay (ELISA)

To determine the plasma level of rat BNP, blood samples from the RV were immediately centrifuged at 3000 rpm at 4°C for 15 minutes. Plasma samples were collected and stored at −80°C. These samples were analyzed using a commercial ELISA kit (Shanghai DoBio Biotech Co. Ltd., Shanghai, China) according to the manufacturer’s instructions.

### Western Blotting

All cardiac proteins were extracted and Western blot analysis was performed as described previously [25]. Frozen RV tissues taken from rats 1 week after MCT injection were weighed, homogenized in ice-cold lysis buffer, and quantified for protein levels using a commercial assay (KeyGen Biotech). One hundred milligrams of protein from each sample was loaded onto 15% SDS-PAGE gel and subjected to electrophoresis using a constant voltage. The proteins were transferred to polyvinylidence fluoride (PVDF) membrane (Millipore, Billerica, MA, U.S.). The membrane was incubated with rabbit anti-Bcl-2 monoclonal antibody, mouse anti-Bax monoclonal antibody (Cell Signaling Technology, Beverly, MA, U.S.), and glyceraldehyde-6-phosphate dehydrogenase (GAPDH, Santa Cruz Biotechnology, Santa Cruz, CA, U.S.) overnight at 4°C. After washing, the membranes were incubated for 1 hour with appropriate horseradish peroxidase-conjugated secondary antibodies. GAPDH was used as loading control. Then protein bands were detected by enhanced chemiluminescence (ECL, Cell Signaling Technology) and visualized using VersaDoc Imaging System (Bio-Rad, Hercules, CA, U.S.). The intensities of the bands were analyzed densitometrically using NIH Image 1.46 software.

### Statistical Analysis

All data in tables and figures are shown in terms of the mean ± SD. Data were subjected to statistical analysis using SPSS 15.0 software (SPSS, Chicago, IL, U.S.). Comparisons between groups were made using one-way ANOVA and the least significant difference (LSD). *P* values of less than 0.05 were considered significant.

## Results

### Nicorandil Attenuated MCT-induced Growth Retardation and Mortality

**Figure 1:**
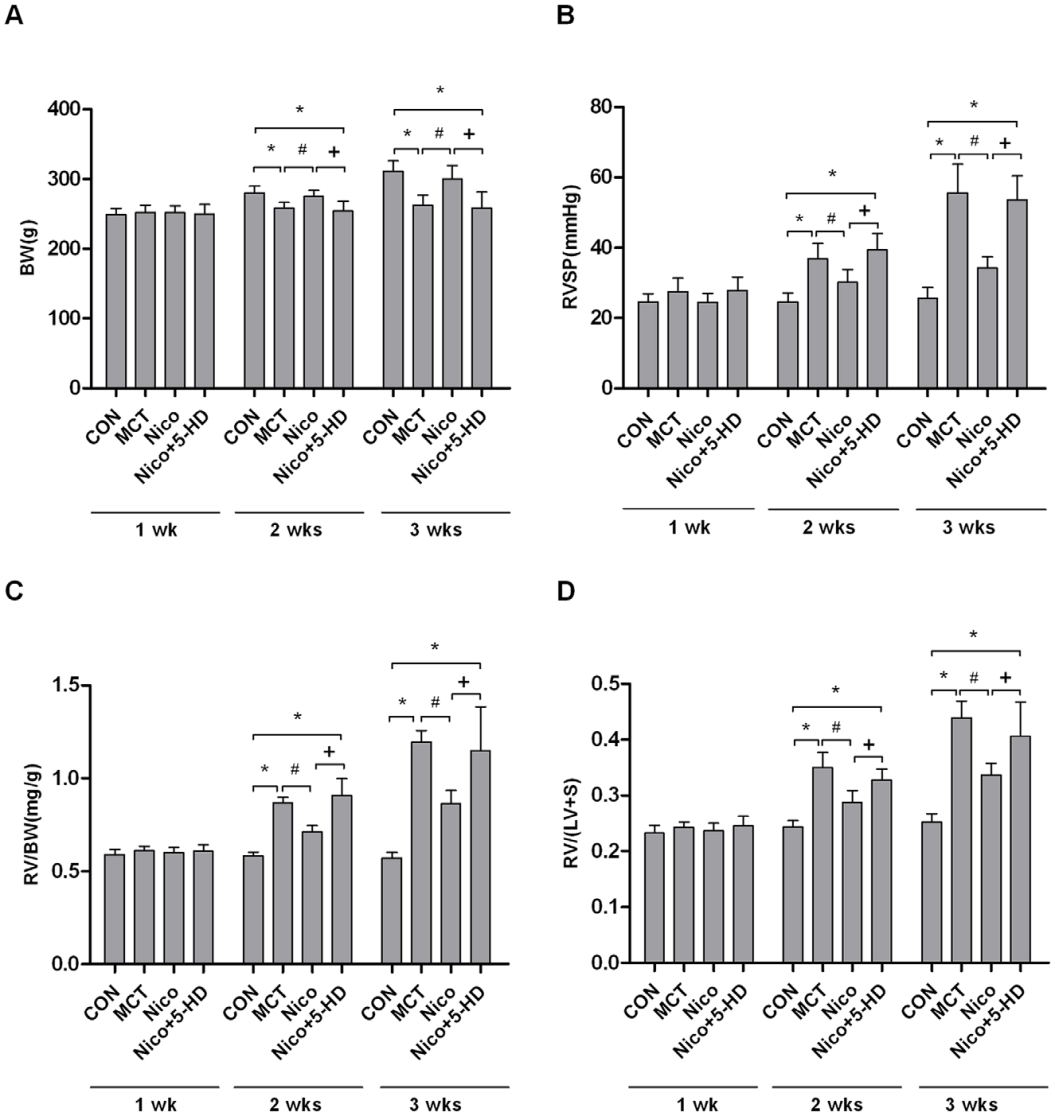
Effects of nicorandil on body weight, right ventricle systolic pressures and hypertrophy in rats on weeks 1–3 after monocrotaline administration. Measurements for the (A) BW, (B) RVSP, (C) RV/BW ratio, and (D) RV/(LV+S) ratio are presented as mean ± SD. BW, body weight; RVSP, right ventricle systolic pressures; MCT, monocrotaline. RV/BW represents the ratio of RV weight to BW, and RV/[LV+S] represents the ratio of the RV weight to left ventricular(LV) plus septum (S) weight. These are indicators of RV hypertrophy. Male SD rats were given a single dose (60 mg/kg) of MCT intraperitoneally and then fed 7.5 mg·kg^−1^·d^−1^ nicorandil (nicorandil-treated group), 5 mg·kg^−1^·d^−1^ 5-hydroxydecanoate (5-HD) combined with 7.5 mg·kg^−1^·d^−1^ nicorandil (nicorandil+5-HD-treated group), or saline (MCT-treated group) by oral gavage from day 1 to day 21. The age- and weight-matched control animals were injected with an equal volume of saline (control group) intraperitoneally, and then given saline for 21 d. CON represents the control group, MCT represents the MCT-treated group, Nico represents the nicorandil-treated group, Nico+5-HD group represents the nicorandil+5-HD-treated group. n = 8 for each group except for n = 7 in Nico+5-HD group at the second week, n = 6 in MCT group and n = 5 in Nico+5-HD group at third week after MCT injection. *P<0.05 compared with control group, #*P*<0.05 relative to the MCT-treated group. +*P*<0.05 relative to the nicorandil-treated group.

There was no significant difference in BW among the experimental groups on days 0 and 7. However, the BW of MCT-treated rats were significantly lower than those of age-matched saline-treated control animals on days 14 and 21 (11.07% and 15.43%, *P*<0.05). Daily nicorandil treatment over the course of 3 weeks attenuated MCT-induced reductions in growth (BW values were 10.44% and 14.07% higher than those of rats treated with MCT at weeks 2 and 3, respectively) and this effect was abolished by 5-HD (Figure 1A). These results are in accordance with the literature [26], [27], [28].

Hongo et al. found that nicorandil improves survival in MCT-induced PAH rats [16]. During the 3-week treatment period and the following period of anesthesia, two rats from the MCT-treated group died, 4 from the Nico+5-HD-treated group died, and none from the control group or nicorandil-treated group died. Although our study was not designed to be a mortality study, these data also suggest that nicorandil reduced the death rate in the MCT-treated group. Consequently, at the end of week 2, hemodynamic measurements could be performed on only 7 rats from the Nico+5-HD-treated group. At week 3, there were only 6 in the MCT group and 5 in the Nico+5-HD-treated group.

### Nicorandil Decreased MCT-induced Elevation of RVSP Levels

To determine the changes of hemodynamic parameters over the course of the development of MCT-induced PAH, we examined rats at the end of each week after injection with MCT and compared them to control animals. Mean blood pressure and heart rate did not differ significantly among the groups (data not shown). Compared to the controls, MCT-injected rats had higher RVSP. This appeared as early as the first week after injection, and showed a significant increase during the second week, which continued into the third week (MCT: 27.54±3.92 mmHg, 36.90±4.34 mmHg, 55.57±8.22 mmHg for weeks 1, 2, and 3 respectively vs. Control: 24.61±3.0 mmHg, 24.53±2.60 mmHg, 25.65±3.04 mmHg) (Figure 1B). Nicorandil decreased RVSP by 11.37%, 17.99%, and 38.29% in MCT-treated rats in weeks 1, 2, and 3, respectively. These effects of nicorandil were found to be blocked by 5-HD (Figure 1B).

### Nicorandil Inhibited MCT-induced RVH

**Figure 2:**
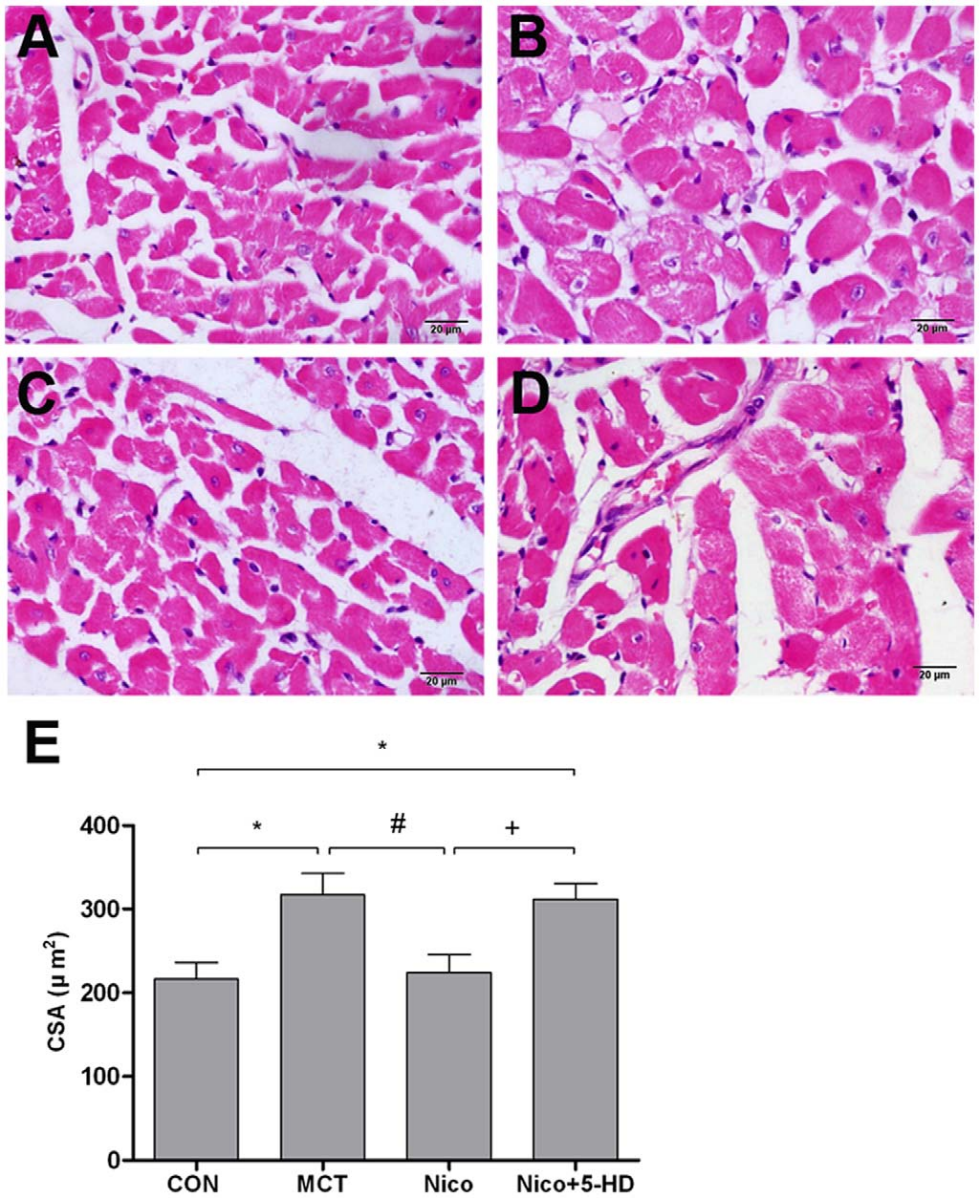
Effects of nicorandil on RV cardiomyocyte hypertrophy in rats with MCT-induced pulmonary arterial hypertension on day 21. Representative photomicrographs (HE stain) of cross-sections of RV myocytes from the following experimental groups: (A) Control group, (B) MCT-treated group, (C) nicorandil-treated group, and (D) nicorandil+5-HD-treated group. Abbreviations are the same as those given in Figure 1. Original magnification, ×400, scale bar = 20 µm. (E) Quantitative analysis of mean cross sectional area (CSA) of RV cardiomyocytes in rats. Abbreviations are the same as those used in Figure 1. Details of treatment groups are described in the legend of Figure 1. Data are presented as mean ± SD (n = 5). *P<0.05 relative to the control group, #*P*<0.05 relative to the MCT-treated group. +*P*<0.05 relative to the nicorandil-treated group.

Chronic RV pressure overload secondary to PAH resulted in RVH, as indicated by RV/BW and RV/[LV+S] ratios in MCT rats. As shown in Figures 1C and 1D, RV/BW, and RV/[LV+S] were significantly higher in the MCT-treated group than in the control group on days 14 and 21. Nicorandil significantly decreased RV/BW (0.71±0.03 and 0.86±0.07 for the nicorandil group at weeks 2 and 3, respectively, vs. 0.90±0.03 and 1.19±0.06 for the MCT-treated group at the same times, *P*<0.05) and RV/[LV+S] (0.29±0.02 and 0.34±0.02 for the nicorandil group at weeks 2 and 3, respectively vs. 0.33±0.02 and 0.40±0.06 for the MCT-treated group). 5-HD inhibited the effect of nicorandil on RVH (Figures 1C and 1D), indicating that 5-HD inhibited the effect of nicorandil completely. RVH was determined by measuring the mean CSA of RV cardiomyocytes. HE staining of the RV tissues showed that the CSA of cardiomyocytes in the MCT group was significantly greater than in the control group at day 21 (Figures 2A, 2B, and 2E *P*<0.05). Treatment with nicorandil at 7.5 mg/kg prevented the increase in the CSA of cardiomyocytes induced by MCT (Figure 2C). This change was inhibited by chronic administration of 5-HD as the values of the CSA of cardiomyocytes approached those of the MCT group (Figure 2D).

### Nicorandil Diminished the Extent of Fibrosis Induced by MCT

**Figure 3:**
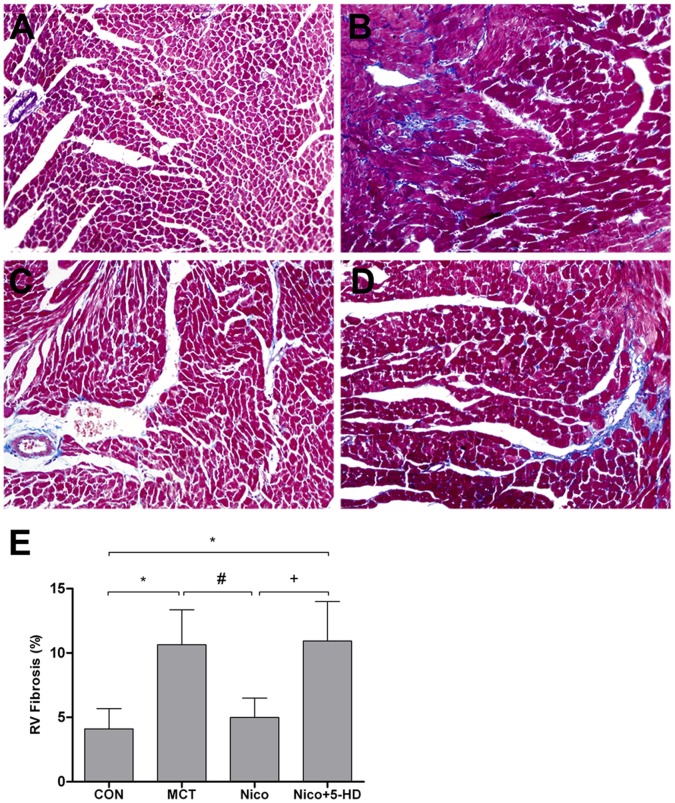
Effects of nicorandil on MCT-induced cardiomyocyte fibrosis in rat RV, 3 weeks after MCT injection. Representative Masson’s Trichrome staining of cardiomyocyte sections showing the extent of interstitial and perivascular fibrosis in the RV-free walls for the following experimental groups: (A) Control group, (B) MCT-treated group, (C) nicorandil-treated group, and (D) nicorandil+5-HD-treated group. Original magnification, ×200. (E) Quantification of fibrosis in the stained cardiomyocyte sections (blue-stained areas expressed as percentage of total RV surface area) from CON, MCT, Nico, and Nico+5-HD groups. Abbreviations are the same as those used in Figure 1. Details of treatment groups are given in the legend of Figure 1. Data are presented as mean ± SD (n = 5). *P<0.05 relative to the control group, #*p*<0.05 relative to the MCT-treated group. +*P*<0.05 relative to the nicorandil-treated group.

RV remodeling is associated with both variations in the morphology of the cardiomyocytes and with increased interstitial fibrosis (increased myocardial collagen content). As demonstrated by Masson’s Trichrome stain, significant interstitial and perivascular fibrosis was observed in the hypertrophic right-side hearts induced by MCT, while nicorandil treatment diminished the extent of fibrosis (Figure 3). However, the suppressive effects of nicorandil were reversed by 5-HD. These results suggest that 5-HD abolishes the inhibition of nicorandil in the case of interstitial fibrosis.

### Nicorandil Restored MCT-induced Ultrastructural Abnormalities in RV

**Figure 4:**
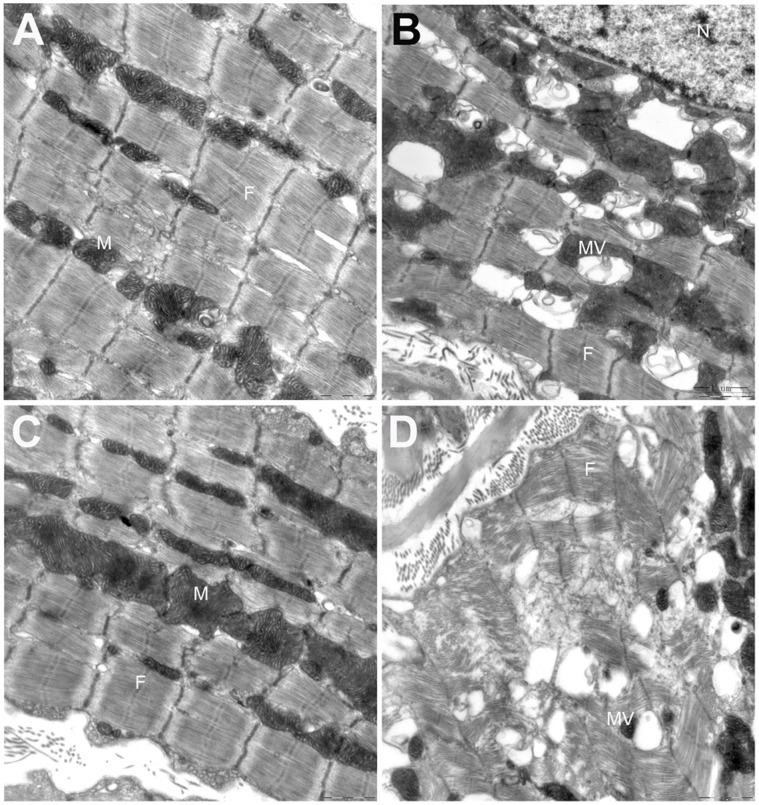
Effects of nicorandil on the ultrastructural changes in RV myocardial cells of MCT-induced pulmonary arterial hypertension rats. (A) The control group showed normal myocyte structure. (B) The MCT-treated group showed swelling and vacuolization of mitochondria, dilatation of the sarcoplasmic reticulum, dissolution of the myofilaments, and an irregular pattern of transverse striations not observed in the control group; (C) The nicorandil-treated group showed intact myocyte structure, suggesting that nicorandil ameliorates MCT-induced myocardial damage. (D) The nicorandil+5-HD-treated group showed disrupted myocyte ultrastructure similar to that observed in the MCT group, which suggests that 5-HD reversed the inhibitory effects of nicorandil. Details of treatment groups are given in legend of the Figure 1. M, mitochondria; N, nucleus; F, myofilaments; MV, mitochondrial vacuolization. Scale bar = 1 µm.

Transmission electron microscopy of the RV cardiomyocytes revealed abnormalities in the MCT-treated group not visible in the control group. These included such as increased mitochondrial density, swelling, vacuolization, and medullary sheath-like degeneration, enlarged sarcoplasmic reticulum, dissolution of the myofilaments, broken Z-lines, and an irregular pattern of transverse striations (Figures 4A and 4B). Treatment with nicorandil prevented these abnormalities (Figure 4C), and treatment with the selective mitoK_ATP_ channel blocker 5-HD reversed the protective effects of nicorandil (Figure 4D).

### Nicorandil Inhibited MCT-induced Cardiomyocyte Apoptosis in the RV

**Figure 5:**
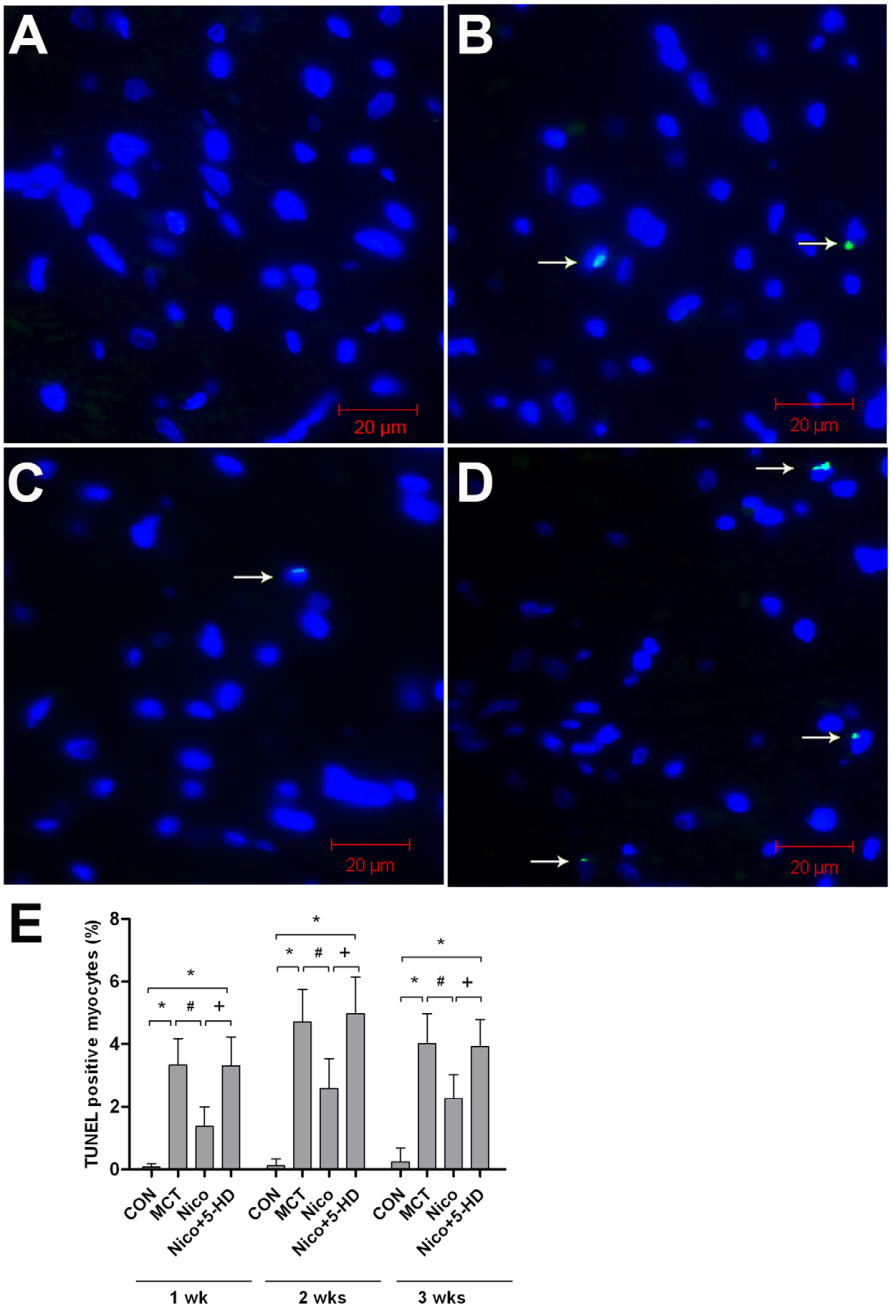
Effects of nicorandil on cardiac myocyte apoptosis in the RV myocardium. Apoptotic cardiomyocytes were detected by terminal deoxyribonucleotidyl transferase-mediated dUTP-digoxigenin nick end-labeling (TUNEL) staining in RV tissue from rats. Green fluorescence shows TUNEL-positive nuclei; blue fluorescence shows nuclei of total cardiomyocytes. (A–D) Representative photomicrographs of TUNEL-stained cells (white arrow) in RV at the first week after MCT injection. (E) Quantitative analysis of TUNEL-positive cardiomyocytes as the percentage of total cell number in right ventricles of the CON, MCT, Nico, and Nico+5-HD groups at 1–3 weeks post-MCT injection. Abbreviations are the same as those used in Figure 1. Representative photomicrographs of TUNEL-stained cells in RV in different groups at the second and third week after MCT injection are not shown. Details of treatment groups are given in the legend of Figure 1. Data are presented as mean ± SD (n = 4). *P<0.05 relative to the control group, #*P*<0.05 relative to the MCT-treated group. +*P*<0.05 relative to the nicorandil-treated group. Scale bars = 20 µm.

There were markedly more TUNEL-positive cardiomyocytes in the RV in the MCT-treated group than in the control group. This difference was observed as early as 1 week post MCT-injection and continued into week 3 (Figures 5A and 5E). Photomicrographs of TUNEL-stained cells in RV in different groups during the second and third weeks after MCT injection are not shown. As shown in Figure 5C, nicorandil significantly inhibited cardiomyocyte apoptosis in the RV during the first week after MCT injection (*P*<0.05 vs MCT; Figures 5B and 5C). In contrast, treatment with 5-HD in combination with nicorandil resulted in TUNEL-positive cardiomyocytes in the RV, similar in number to time-matched MCT-treated rats. This demonstrates that 5-HD blocked the effect of nicorandil on apoptosis of cardiomyocytes induced by MCT (Figure 5D).

### Nicorandil Suppressed the MCT-induced Increase in Plasma BNP

**Figure 6:**
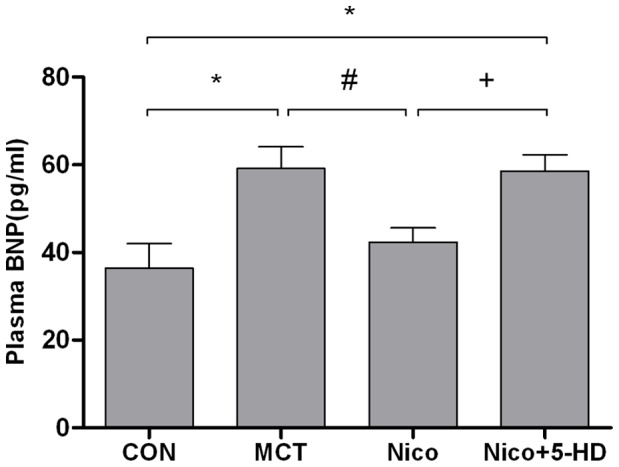
Effects of nicorandil on plasma levels of BNP in MCT- induced pulmonary arterial hypertension rats. Results of an enzyme-linked immunosorbent assay analysis of plasma BNP concentration from CON (n = 8), MCT (n = 6), Nico (n = 8), and Nico+5-HD (n = 5) groups. Abbreviations are the same as those used in Figure 1. Details of treatment groups are described in the legend of Figure 1. Data are presented as mean ± SD. *P<0.05 relative to the control group, #*P*<0.05 relative to the MCT-treated group. +*P*<0.05 relative to the nicorandil-treated group.

Plasma BNP is used for the diagnosis and staging of heart failure [29], because its levels become elevated in proportion to the degree of RV dysfunction and RV remodeling in PAH [30], [31], [32], [33], [34]. At 3 weeks, plasma BNP levels were markedly higher in MCT-injected animals than in age-matched control animals (59.16±4.96 pg/ml vs. 36.43±5.58 pg/ml). This increase was significantly suppressed by nicorandil. 5-HD also prevented the decrease in plasma BNP level induced by nicorandil (Figure 6).

### Nicorandil Partially Reversed the MCT-induced Decrease in the Bcl-2/Bax Ratio

**Figure 7:**
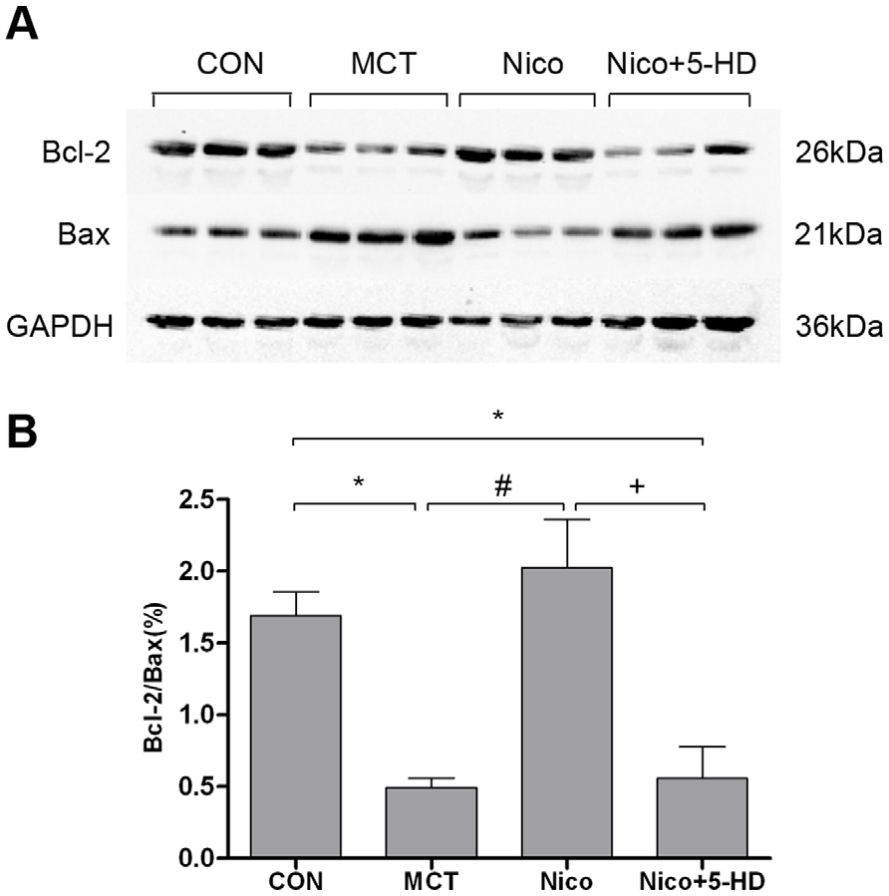
Effects of nicorandil on Bcl-2 and Bax protein expression in the RV, 1 week after MCT injection. The protein levels of Bcl-2 and Bax in the RV myocardium were determined by Western blot analysis. The levels of Bcl-2 and Bax protein were quantitatively analyzed with an image analyzer and expressed as the ratio of Bcl-2/Bax. Equal loading of protein was confirmed with anti-GAPDH antibody. (A) Representative Western blot results showing the expression of Bcl-2, Bax, and GAPDH protein in the right ventricle of rats from different experimental groups. (B) Bar graph showing the densitometric scanning of the ratio of Bcl-2/Bax. Abbreviations are the same as in the legend of Figure 1. Data are presented as mean ± SD (n = 3). *P<0.05 relative to the control group, #*P*<0.05 compared with MCT-treated group. +*P*<0.05 relative to the nicorandil-treated group.

Western blotting was performed to examine the role of nicorandil on Bcl-2 and Bax in rat right heart tissue at week 1. As shown in Figure 7, the Bcl-2/Bax ratio was significantly lower in the MCT-injected animals than in controls at week 1 (*P*<0.05). Nicorandil partially reversed the decrease in the Bcl-2/Bax ratio at week 1 (*P*<0.05), whereas co-treatment with nicorandil and 5-HD decreased the ratio to the level observed in the MCT-injected rats (Figure 7).

## Discussion

In this study, we successfully established an RV remodeling model *in vivo*. We induced PAH in rats using MCT for three weeks, and these rats displayed cardiac hypertrophy, fibrosis, and dysfunction consistent with previous studies [25], [35], [36]. We also demonstrated for the first time that nicorandil effectively attenuated the PAH-induced RV remodeling. This effect was probably associated not only with a reduction in RVSP but also inhibition of RV apoptosis.

In this study, injecting MCT (60 mg/kg, ip) into rats resulted in RV remodeling, as previously reported [25], [35], [36]. In MCT-injected rats, RVSP gradually increased from 24.61±3.0 mmHg at baseline to 36.90±4.34 mmHg at 2 weeks and 55.57±8.22 mmHg at 3 weeks. These results are consistent with the findings of previous studies in that MCT-induced PAH in rats is apparent by day 14 and worsens by day 21 (Figure 1B) [24], [37], [38]. The progression of RVH is indicated by the increased RV/BW and RV/[LV+S] ratios due to local pressure overload after 2 weeks [26], [27], [39]. The RV/BW ratio had a 108.77% increase and the RV/[LV+S] a 76.00% increase during the third week. This process was accompanied by increased CSA in RV cardiomyocytes (Figure 2B) and remarkable interstitial fibrosis in RV (Figure 3B). Plasma BNP levels increased by 62.39% in MCT-induced PAH rats. All of these observations strongly indicate that MCT can induce the formation of RV remodeling and dysfunction [25], [35], [36].

Nicorandil can ameliorate MCT-induced PAH in rats, possibly by significantly decreasing RV afterload and hypertrophy and by improving pulmonary artery remodeling [16], [40]. Consistent with this, we found that nicorandil significantly decreased RVSP, RV/BW, RV/[LV+S], BNP, and interstitial fibrosis relative to the MCT group. In this way, we confirmed that nicorandil can improve RV remodeling and dysfunction induced by PAH.

Elevated PAP (RV afterload) is the main cause of PAH-induced RV maladaptive hypertrophy and failure. Recently, Bogaard et al. stated that an elevated PAP is not the only reason for PAH-associated RV remodeling and failure [12]. Apoptosis of the cardiomyocytes may also play a role in RV remodeling and in the subsequent development of heart failure [12], [41], [42], [43]. Previous studies suggest that cardiomyocyte apoptosis is implicated in RV remodeling after acute left ventricular myocardial infarction [44] and pulmonary arterial banding [45], [46]. However, the role of cardiomyocyte apoptosis in the development of PAH-induced RV remodeling and failure is still unknown. Maria et al. showed that apoptosis associated with fibrosis and capillary rarefaction is involved in RV hypertrophy, dilation, and failure in an angioproliferative pulmonary hypertension rat model, induced by combined exposure to the VEGF receptor blocker SU5416 and hypoxia [15]. Apoptosis is observed early and then declined when clinically manifest RV failure occurs. This may provide new insight into the time course of cardiac cell apoptosis in RV remodeling and aid in determining the optimal timing of antiapoptotic therapy to prevent or reverse RV remodeling. Theoretically, any drug capable of reducing both PAP and RV remodeling would be promising for the treatment of PAH. In the present study, our results show that in the MCT-induced cardiac remodeling rat model, there were significantly more apoptotic cardiomyocytes than in the control group. Apoptosis was observed as early as the first week after MCT treatment, even though there was no obvious elevation in RVSP. This indicated that early apoptosis might be involved in RV remodeling except for pressure overload. Nicorandil was found to reduce the number of apoptotic cardiomyocytes remarkably from the first week to the third week, in parallel to the reduction of RVH. This anti-apoptotic effect may contribute to the amelioration of MCT-induced RV remodeling.

Mitochondria are considered the central regulators of apoptosis [47], and play a vital role in cardiac remodeling and failure [48], [49]. It is well established that the Bcl-2/Bax ratio determines cell apoptotic fate [50], [51]. In our study, the Bcl-2/Bax ratio was reduced significantly (*P*<0.05) in the right myocardium 1 week after MCT injection compared with the control group (Figure 7). Apoptosis was detected by TUNEL assay as early as 1 week after MCT injection. This was consistent with the dramatic down-regulation of Bcl-2/Bax. This supports the hypothesis that apoptosis might be involved in a critical dysregulatory mechanism in the maladaptive cardiac response during the progression of pulmonary hypertension.

Many studies have demonstrated that mitoK_ATP_ channels are implicated in the mechanism of cell apoptosis [52]. Nicorandil, through its activation of mitoK_ATP_ channels, can inhibit apoptosis in cardiomyocytes by improving mitochondrial function, preventing calcium overload in the mitochondria, and preserving mitochondrial integrity [17], [18], [19], [53]. In this study, nicorandil not only significantly reduced MCT-induced ultrastructural injuries to the mitochondria but also inhibited cardiomyocyte apoptosis in the RV as measured by the increase in the relative expression of Bcl-2/Bax in mitochondria. The anti-apoptotic effects of nicorandil may in part contribute to the amelioration of MCT-induced myocardial injuries and RV remodeling.

In conclusion, our results clearly show that nicorandil, a mitoK_ATP_ channel opener, can inhibit MCT-induced RV remodeling in rats. This effect is mediated not only by reduction of RVSP but also by inhibition of apoptotic cell death. Nicorandil can reduce mitochondrial impairment and inhibit the alteration of Bcl-2 and Bax in the mitochondria of cardiomyocytes in the MCT-treated rats. Its protective effect in PAH- induced RV remodeling may involve opening of the mitoK_ATP_ channel. For this reason, we believe that the use of a mitoK_ATP_ channel opener such as nicorandil may represent a new therapeutic strategy for ameliorating RV remodeling during the early stages of PAH.

## References

[1] McLaughlin VV, Archer SL, Badesch DB, Barst RJ, Farber HW, et al (2009) ACCF/AHA 2009 expert consensus document on pulmonary hypertension a report of the American College of Cardiology Foundation Task Force on Expert Consensus Documents and the American Heart Association developed in collaboration with the American College of Chest Physicians; American Thoracic Society, Inc. and the Pulmonary Hypertension Association. J Am Coll Cardiol 53: 1573–1619. 19389575 10.1016/j.jacc.2009.01.004

[2] Rhodes CJ, Davidson A, Gibbs JS, Wharton J, Wilkins MR (2009) Therapeutic targets in pulmonary arterial hypertension. Pharmacol Ther 121: 69–88. 19010350 10.1016/j.pharmthera.2008.10.002

[3] D'Alonzo GE, Barst RJ, Ayres SM, Bergofsky EH, Brundage BH, et al (1991) Survival in patients with primary pulmonary hypertension. Results from a national prospective registry. Ann Intern Med 115: 343–349. 1863023 10.7326/0003-4819-115-5-343

[4] van Wolferen SA, Marcus JT, Boonstra A, Marques KM, Bronzwaer JG, et al (2007) Prognostic value of right ventricular mass, volume, and function in idiopathic pulmonary arterial hypertension. Eur Heart J 28: 1250–1257. 17242010 10.1093/eurheartj/ehl477

[5] Jing ZC, Xu XQ, Han ZY, Wu Y, Deng KW, et al (2007) Registry and survival study in chinese patients with idiopathic and familial pulmonary arterial hypertension. Chest 132: 373–379. 17400671 10.1378/chest.06-2913

[6] Vonk Noordegraaf A, Naeije R (2008) Right ventricular function in scleroderma-related pulmonary hypertension. Rheumatology (Oxford) 47 Suppl 5v42–43. 18784141 10.1093/rheumatology/ken284

[7] Humbert M, Sitbon O, Chaouat A, Bertocchi M, Habib G, et al (2006) Pulmonary arterial hypertension in France: results from a national registry. Am J Respir Crit Care Med 173: 1023–1030. 16456139 10.1164/rccm.200510-1668OC

[8] Voelkel NF, Quaife RA, Leinwand LA, Barst RJ, McGoon MD, et al (2006) Right Ventricular Function and Failure: Report of a National Heart, Lung, and Blood Institute Working Group on Cellular and Molecular Mechanisms of Right Heart Failure. Circulation 114: 1883–1891. 17060398 10.1161/CIRCULATIONAHA.106.632208

[9] Bogaard HJ, Natarajan R, Mizuno S, Abbate A, Chang PJ, et al (2010) Adrenergic receptor blockade reverses right heart remodeling and dysfunction in pulmonary hypertensive rats. Am J Respir Crit Care Med 182: 652–660. 20508210 10.1164/rccm.201003-0335OC

[10] Simon MA, Pinsky MR (2011) Right ventricular dysfunction and failure in chronic pressure overload. Cardiol Res Pract 2011: 568095. 21559218 10.4061/2011/568095 PMC3087982

[11] Dore A, Houde C, Chan KL, Ducharme A, Khairy P, et al (2005) Angiotensin receptor blockade and exercise capacity in adults with systemic right ventricles: a multicenter, randomized, placebo-controlled clinical trial. Circulation 112: 2411–2416. 16216961 10.1161/CIRCULATIONAHA.105.543470

[12] Bogaard HJ, Natarajan R, Henderson SC, Long CS, Kraskauskas D, et al (2009) Chronic Pulmonary Artery Pressure Elevation Is Insufficient to Explain Right Heart Failure. Circulation 120: 1951–1960. 19884466 10.1161/CIRCULATIONAHA.109.883843

[13] Voelkel NF, Natarajan R, Drake JI, Bogaard HJ (2011) Right Ventricle in Pulmonary Hypertension. Comprehensive Physiology: John Wiley & Sons, Inc. p.525–540. 10.1002/cphy.c090008 23737184

[14] Dorn GW (2008) Apoptotic and non-apoptotic programmed cardiomyocyte death in ventricular remodelling. Cardiovascular Research 81: 465–473. 18779231 10.1093/cvr/cvn243 PMC2721651

[15] Campian ME, Verberne HJ, Hardziyenka M, de Bruin K, Selwaness M, et al (2009) Serial Noninvasive Assessment of Apoptosis During Right Ventricular Disease Progression in Rats. Journal of Nuclear Medicine 50: 1371–1377. 19617336 10.2967/jnumed.108.061366

[16] Hongo M, Mawatari E, Sakai A, Ruan Z, Koizumi T, et al (2005) Effects of nicorandil on monocrotaline-induced pulmonary arterial hypertension in rats. J Cardiovasc Pharmacol 46: 452–458. 16160596 10.1097/01.fjc.0000176728.74690.09

[17] Nagata K (2003) Nicorandil inhibits oxidative stress-induced apoptosis in cardiac myocytes through activation of mitochondrial ATP-sensitive potassium channels and a nitrate-like effect. Journal of Molecular and Cellular Cardiology 35: 1505–1512. 14654376 10.1016/j.yjmcc.2003.09.018

[18] Nishikawa S, Tatsumi T, Shiraishi J, Matsunaga S, Takeda M, et al (2006) Nicorandil regulates Bcl-2 family proteins and protects cardiac myocytes against hypoxia-induced apoptosis. Journal of Molecular and Cellular Cardiology 40: 510–519. 16527305 10.1016/j.yjmcc.2006.01.020

[19] Akao M, Teshima Y, Marban E (2002) Antiapoptotic effect of nicorandil mediated by mitochondrial atp-sensitive potassium channels in cultured cardiac myocytes. J Am Coll Cardiol 40: 803–810. 12204514 10.1016/s0735-1097(02)02007-7

[20] Lee T-M, Lin M-S, Chang N-C (2008) Effect of ATP-Sensitive Potassium Channel Agonists on Ventricular Remodeling in Healed Rat Infarcts. Journal of the American College of Cardiology 51: 1309–1318. 18371564 10.1016/j.jacc.2007.11.067

[21] Saeed M, Watzinger N, Krombach GA, Lund GK, Wendland MF, et al (2002) Left ventricular remodeling after infarction: sequential MR imaging with oral nicorandil therapy in rat model. Radiology 224: 830–837. 12202722 10.1148/radiol.2243011372

[22] Sanada S, Node K, Asanuma H, Ogita H, Takashima S, et al (2002) Opening of the adenosine triphosphate-sensitive potassium channel attenuates cardiac remodeling induced by long-term inhibition of nitric oxide synthesis: role of 70-kDa S6 kinase and extracellular signal-regulated kinase. J Am Coll Cardiol 40: 991–997. 12225728 10.1016/s0735-1097(02)02057-0

[23] Eguchi Y, Takahari Y, Higashijima N, Ishizuka N, Tamura N, et al (2009) Nicorandil attenuates FeCl(3)-induced thrombus formation through the inhibition of reactive oxygen species production. Circ J 73: 554–561. 19179768 10.1253/circj.cj-08-0843

[24] Hardziyenka M, Campian ME, de Bruin-Bon HA, Michel MC, Tan HL (2006) Sequence of echocardiographic changes during development of right ventricular failure in rat. J Am Soc Echocardiogr 19: 1272–1279. 17000367 10.1016/j.echo.2006.04.036

[25] Okada M, Harada T, Kikuzuki R, Yamawaki H, Hara Y (2009) Effects of telmisartan on right ventricular remodeling induced by monocrotaline in rats. J Pharmacol Sci 111: 193–200. 19809219 10.1254/jphs.09112fp

[26] Miyauchi T, Yorikane R, Sakai S, Sakurai T, Okada M, et al (1993) Contribution of endogenous endothelin-1 to the progression of cardiopulmonary alterations in rats with monocrotaline-induced pulmonary hypertension. Circ Res 73: 887–897. 8403258 10.1161/01.res.73.5.887

[27] Buermans HP, Redout EM, Schiel AE, Musters RJ, Zuidwijk M, et al (2005) Microarray analysis reveals pivotal divergent mRNA expression profiles early in the development of either compensated ventricular hypertrophy or heart failure. Physiol Genomics 21: 314–323. 15728335 10.1152/physiolgenomics.00185.2004

[28] Honda M, Yamada S, Goto Y, Ishikawa S, Yoshikane H, et al (1992) Biochemical and structural remodeling of collagen in the right ventricular hypertrophy induced by monocrotaline. Jpn Circ J 56: 392–403. 1533690 10.1253/jcj.56.392

[29] de Lemos JA, McGuire DK, Drazner MH (2003) B-type natriuretic peptide in cardiovascular disease. Lancet 362: 316–322. 12892964 10.1016/S0140-6736(03)13976-1

[30] Nagaya N, Nishikimi T, Uematsu M, Satoh T, Kyotani S, et al (2000) Plasma brain natriuretic peptide as a prognostic indicator in patients with primary pulmonary hypertension. Circulation 102: 865–870. 10952954 10.1161/01.cir.102.8.865

[31] Nagaya N, Nishikimi T, Okano Y, Uematsu M, Satoh T, et al (1998) Plasma brain natriuretic peptide levels increase in proportion to the extent of right ventricular dysfunction in pulmonary hypertension. J Am Coll Cardiol 31: 202–208. 9426041 10.1016/s0735-1097(97)00452-x

[32] Shiina Y, Funabashi N, Lee K, Daimon M, Sekine T, et al (2009) Right atrium contractility and right ventricular diastolic function assessed by pulsed tissue Doppler imaging can predict brain natriuretic peptide in adults with acquired pulmonary hypertension. Int J Cardiol 135: 53–59. 18793807 10.1016/j.ijcard.2008.03.090

[33] Yap LB (2004) B-type natriuretic Peptide and the right heart. Heart Fail Rev 9: 99–105. 15516857 10.1023/B:HREV.0000046364.68371.b0

[34] Reesink HJ, Tulevski, II, Marcus JT, Boomsma F, Kloek JJ, et al (2007) Brain natriuretic peptide as noninvasive marker of the severity of right ventricular dysfunction in chronic thromboembolic pulmonary hypertension. Ann Thorac Surg 84: 537–543. 17643631 10.1016/j.athoracsur.2007.04.006

[35] Hessel MH, Steendijk P, den Adel B, Schutte CI, van der Laarse A (2006) Characterization of right ventricular function after monocrotaline-induced pulmonary hypertension in the intact rat. Am J Physiol Heart Circ Physiol 291: H2424–2430. 16731643 10.1152/ajpheart.00369.2006

[36] Lourenco AP, Roncon-Albuquerque R Jr, Bras-Silva C, Faria B, Wieland J, et al (2006) Myocardial dysfunction and neurohumoral activation without remodeling in left ventricle of monocrotaline-induced pulmonary hypertensive rats. Am J Physiol Heart Circ Physiol 291: H1587–1594. 16679394 10.1152/ajpheart.01004.2005

[37] Henkens IR, Mouchaers KT, Vliegen HW, van der Laarse WJ, Swenne CA, et al (2007) Early changes in rat hearts with developing pulmonary arterial hypertension can be detected with three-dimensional electrocardiography. Am J Physiol Heart Circ Physiol 293: H1300–1307. 17496210 10.1152/ajpheart.01359.2006

[38] Handoko ML, Schalij I, Kramer K, Sebkhi A, Postmus PE, et al (2008) A refined radio-telemetry technique to monitor right ventricle or pulmonary artery pressures in rats: a useful tool in pulmonary hypertension research. Pflugers Arch 455: 951–959. 17912547 10.1007/s00424-007-0334-z PMC2137943

[39] Lee JK, Nishiyama A, Kambe F, Seo H, Takeuchi S, et al (1999) Downregulation of voltage-gated K(+) channels in rat heart with right ventricular hypertrophy. Am J Physiol 277: H1725–1731. 10564125 10.1152/ajpheart.1999.277.5.H1725

[40] Sahara M, Sata M, Morita T, Hirata Y, Nagai R (2012) Nicorandil attenuates monocrotaline-induced vascular endothelial damage and pulmonary arterial hypertension. PLoS One 7: e33367. 22479390 10.1371/journal.pone.0033367 PMC3316574

[41] Kret M, Arora R (2007) Pathophysiological basis of right ventricular remodeling. J Cardiovasc Pharmacol Ther 12: 5–14. 17495253 10.1177/1074248406298293

[42] Haworth SG (2007) The cell and molecular biology of right ventricular dysfunction in pulmonary hypertension. European Heart Journal Supplements 9: H10–H16.

[43] Bogaard HJ, Abe K, Vonk Noordegraaf A, Voelkel NF (2009) The Right Ventricle Under Pressure: Cellular and Molecular Mechanisms of Right-Heart Failure in Pulmonary Hypertension. Chest 135: 794–804. 19265089 10.1378/chest.08-0492

[44] Bussani R, Abbate A, Biondi-Zoccai GG, Dobrina A, Leone AM, et al (2003) Right ventricular dilatation after left ventricular acute myocardial infarction is predictive of extremely high peri-infarctual apoptosis at postmortem examination in humans. J Clin Pathol 56: 672–676. 12944550 10.1136/jcp.56.9.672 PMC1770058

[45] Ikeda S, Hamada M, Hiwada K (1999) Cardiomyocyte apoptosis with enhanced expression of P53 and Bax in right ventricle after pulmonary arterial banding. Life Sci 65: 925–933. 10465352 10.1016/s0024-3205(99)00322-7

[46] Braun M (2003) Right ventricular hypertrophy and apoptosis after pulmonary artery banding: regulation of PKC isozymes. Cardiovascular Research 59: 658–667. 14499867 10.1016/s0008-6363(03)00470-x

[47] Mignotte B, Vayssiere JL (1998) Mitochondria and apoptosis. Eur J Biochem 252: 1–15. 9523706 10.1046/j.1432-1327.1998.2520001.x

[48] Tsutsui H, Kinugawa S, Matsushima S (2009) Mitochondrial oxidative stress and dysfunction in myocardial remodelling. Cardiovasc Res 81: 449–456. 18854381 10.1093/cvr/cvn280

[49] Piao L, Marsboom G, Archer SL (2010) Mitochondrial metabolic adaptation in right ventricular hypertrophy and failure. J Mol Med 88: 1011–1020. 20820751 10.1007/s00109-010-0679-1 PMC3031098

[50] Gross A (2001) BCL-2 proteins: regulators of the mitochondrial apoptotic program. IUBMB Life 52: 231–236. 11798037 10.1080/15216540152846046

[51] Adams JM, Cory S (1998) The Bcl-2 protein family: arbiters of cell survival. Science 281: 1322–1326. 9735050 10.1126/science.281.5381.1322

[52] Ardehali H, O'Rourke B (2005) Mitochondrial K(ATP) channels in cell survival and death. J Mol Cell Cardiol 39: 7–16. 15978901 10.1016/j.yjmcc.2004.12.003 PMC2692534

[53] Sanbe A, Marunouchi T, Yamauchi J, Tanonaka K, Nishigori H, et al (2011) Cardioprotective effect of nicorandil, a mitochondrial ATP-sensitive potassium channel opener, prolongs survival in HSPB5 R120G transgenic mice. PLoS ONE 6: e18922. 21541347 10.1371/journal.pone.0018922 PMC3081834

